# Burden of Paediatric Dog Bite Injuries on the Emergency Department of an English Coastal Town

**DOI:** 10.7759/cureus.84307

**Published:** 2025-05-17

**Authors:** Kene Maduemem, Catherine Hey, Kenechi Anakebe, Mustafa Boorenie, Livi Watkinson

**Affiliations:** 1 Pediatric Emergency Medicine, Royal Manchester Children's Hospital, Manchester, GBR; 2 Emergency Medicine, Blackpool Victoria Hospital, Blackpool, GBR

**Keywords:** animal attack, child-dog interaction, dog bites, index of multiple deprivation (imd), trauma and injury prevention, trauma burden, trauma injuries

## Abstract

Background

Dog bite injuries can be life threatening and/or life changing for children and their families. The psychological consequences of these injuries are significant yet challenging to quantify. Prevention of these injuries is crucial. We studied the demographics, mechanism of injury and patient outcome in order to identify preventative strategies during emergency department (ED) presentation.

Methods

A single-centre, retrospective study was conducted in the children's ED of Blackpool Victoria Hospital, United Kingdom. Electronic medical records of patients under 16 years old with dog bite injuries between September 1, 2021 to September 30, 2023 were reviewed. Data collected included patient demographics, events surrounding dog bite injuries, safeguarding concerns, management and outcome. Correlations of variables were analysed using chi-squared test.

Results

One hundred and seventy-two cases presented with dog bite injuries. There was a slight male preponderance (94; 54.7%). Injuries were commonly seen in the first decade of life (109; 63.4%). Most of the injuries occurred indoors (132; 76.7%). Clear provocation preceding the bite injuries was documented in 76 children (44.2%). Common sites of bite injuries were face (88; 51.2%) and distal upper extremities (53; 30.8%). Injuries were commonly caused by dogs familiar to their victims (137; 79.7%). Facial injuries were more associated with familiar dogs (p<.00001). Twenty-five (14.5%) children required tertiary care for their bite injuries. There were inconsistencies in antibiotic prescription for dog bite injuries with variations in duration of therapy.

Conclusions

The burden of dog bite injuries in our ED is evident. Most of the bites were caused by familiar dogs in indoor settings. The study provided incredibly insightful data to guide the development of preventative strategies on a local and national level.

## Introduction

Dog bite injuries constitute an often-underestimated global public health issue. The global estimates of dog bite incidence are unknown; however, World Health Organization suggests that tens of millions of injuries annually are caused by dog bites with children being most at risk [1].

Children between the ages of one and 14 years represent the most vulnerable population [2]. Particularly, children under the age of two and those aged 9-12 years are most frequently victims [3]. An American study reported 46% of school-aged children had sustained a dog bite injury before primary school [4]. The risk of head and neck injuries is greater in children, contributing to increased morbidity and mortality [1]. During the COVID-19 pandemic lockdowns, there was a reported increase in dog ownership with an increase in dog bite injuries in the Liverpool region of England [5] and other countries [6, 7].

Dog attacks result in serious physical and psychological consequences and even death [8]. There are significant economic implications [9]. In 2022, 10 fatalities were recorded in England and Wales, with four being children [10]. In February 2024, legal controls were introduced on ownership or possession of XL bully type dogs in England and Wales given the breed's disproportionate involvement in case fatalities [10].

More deprived areas in England and Scotland have been associated with a higher risk of dog bites [3, 11]. The 2019 Indices of Multiple Deprivation ranked Blackpool as the area with the most concentrated deprivation in England [12]. Blackpool ranked third most income-deprived of the 316 local authorities in England [13]. None of the 94 neighbourhoods in Blackpool were in the 20% least income-deprived in England. Given the high level of deprivation in Blackpool, the burden of dog bite injuries deserve attention.

We studied the demographics, mechanism of injury and patient outcome of dog bite injuries in order to identify preventative strategies during emergency department (ED) presentation.

This article was previously presented as an oral presentation at the 2024 European Society for Emergency Medicine Annual Conference on October 14, 2024.

## Materials and methods

A retrospective review of the electronic medical records of patients presenting with dog bite injuries between September 1, 2021 and September 30, 2023 was performed. All children and adolescents up to the eve of their 16th birthday were included. Dog strikes and scratches without puncture wounds were excluded. Two authors collated the list of eligible patients from the hospital records. The rest of co-authors independently screened the list for duplications and exclusion criteria. 

The children's ED of Blackpool Victoria Hospital sees an average of 13,000 children per annum. The hospital serves a population of approximately 440,000 residents across Blackpool, Fylde, Wyre and North Lancashire. Being a coastal town, Blackpool attracts millions of seasonal tourists. Plastic and maxillofacial specialist services are provided in another hospital located in Preston city, 23 kilometres from Blackpool.

Ethical approval was exempted due to the nature of retrospective review. The project was deemed a service evaluation and improvement. It was therefore registered with the hospital clinical audit and effectiveness department (ID 00195).

Data collected comprised patient demographics (age, gender), dog characteristics (dog breed), event preceding dog bite (provocative factor, presence of responsible adult/carer, setting of incident), seasonality of dog bite injuries, sites of bite injuries, safeguarding concerns, wound management, antibiotic therapy and outcome of ED attendance (discharge, follow-up, transfer out). Severe injuries were defined as physical injuries requiring tertiary plastic and/or maxillofacial services.

Data were analysed using IBM SPSS version 23.0 (IBM Corp, Armonk, NY). Categorical variables were descriptively represented as frequencies and percentages. Subgroup analysis based on dog familiarity was undertaken for the following variables; site of bite injury and severity of injuries using chi-squared test. A p-value below 0.05 was considered statistically significant.

## Results

One hundred and seventy-two cases over the two-year period were analysed. The median (range) age was seven (0.8-15.9) years. Table 1 descriptively summarizes the demographics of children, events relating to dog bites and wound management. Incident reported to the police was documented in 53.5% of the cases (n=92). Bite injuries occurred in different home settings in 76.7% of the patients (n=132). Children who were holidaying in Blackpool accounted for 35% of the cases (n=14) of the 40 injuries sustained in public areas. Breed of dog was documented in 65.7% of the cases (n=115), with XL bully breed in four cases (family dogs).

**Table 1:** Summary of demographics of children, events relating to the dog bites, and wound management *N=128 children received antibiotics. **Children received one or more treatment options.

Variables	N = 172 (%)
Age	
<1 year	3 (1.7)
1–4 years	49 (29.3)
5–9 years	57 (32.8)
10–15 years	63 (36.2)
Gender	
Male	94 (54.7)
Female	78 (45.3)
Season of the incident	
Autumn	42 (24.4)
Winter	36 (20.9)
Spring	39 (22.7)
Summer	55 (32)
Dog ownership	
Family	75 (43.6)
Family friend	19 (11)
Relatives	29 (17)
Neighbours	14 (8.1)
Unknown/stray	35 (20.3)
Setting of dog bite	
Child’s home	87 (50.6)
Outdoors	40 (23.3)
Relatives’ home	25 (14.5)
Friends’ home	20 (11.6)
Dog provocation preceding bite	
Yes	76 (44.2)
No	73 (42.4)
Unclear	23 (13.4)
Adult present during incident	
Yes	153 (89)
No	4 (2.3)
Not documented	15 (8.7)
Safeguarding concerns	
Yes	5 (2.9)
No	151 (87.8)
Not documented	16 (9.3)
Prescription of antibiotics*	
Co-amoxiclav	95 (74.2)
Doxycycline	1 (0.8)
Topical chloramphenicol	2 (1.6)
Not documented	30 (23.4)
Wound care**	
Cleaning	145 (84.3)
Wound irrigation	55 (32)
Wound dressing	14 (8.1)
Steri-strips	36 (20.9)
Closure with glue	3 (1.7)
Suturing	3 (1.7)
Not documented	27 (15.7)
Outcome	
Discharged without follow-up	130 (75.6)
Discharged with local follow up	16 (9.3)
Admitted locally	1 (0.6)
Transferred to specialist	25 (14.5)

Facial injuries were seen in 51.2% of the patients (n=88). Injuries to the upper limb (hands, forearm) accounted for 30.1% of the cases (n= 53). Other parts of the body were lower limbs (14%; n=24), trunk (3.5%; n=6) and penis (0.6%; n=1). Facial bite injuries were more likely to be inflicted by familiar dogs (X^2^ (1, N = 172) = 27.76, p<.00001). There was no significant correlation between severity of injuries and familiarity of dogs as shown in Table 2.

**Table 2:** Correlations of familiarity of dogs and dog bite injuries X^2^=Chi-squared test.

Variables	Familiar dogs	Unfamiliar dogs	p values
Children with facial injuries	84	4	
Non-facial injuries	53	31	X^2^ (1, N=172) = 28.60, p<.00001
Children with severe bite injuries	22	3	
Non-severe bite injuries	115	32	X^2^ (1, N=172) = 1.26, p<.26

Antibiotics were prescribed in 74.4% of the patients (n=128). Amoxicillin-clavulanic acid was the antibiotic of choice in 74.2% of the cases (n=95). There were variations in duration of therapy prescribed: three days (n=12), five days (n=25), seven days (n=12) and unknown (n=46).

Twenty-five children were transferred for tertiary services (plastics and maxillofacial) in Preston. The characteristics of this cohort are shown in Table 3.

**Table 3:** Cohort of severe dog bite injuries

Variables	N=25 (%)
Males	13 (52)
Setting of dog bite: child’s home	20 (80)
Provocation preceding bite	12 (48)
Documented adult presence	18 (72)
Familiar dog	22 (88)
Facial injuries	22 (88)
Incident logged with police	17 (68)

The majority of these severe injuries were seen in children younger than 10 years (72%; n=18). The breed of dogs responsible for the injuries was documented in 15 cases (Table 4).

**Table 4:** Dog breeds involved in severe injuries *Crossbreed of Labrador and bulldog

Breed of dog	N=15
French bulldog	3
Dachshund	2
German Shepherd	1
Chow Chow	1
Golden Retriever	1
Border Collier	1
Husky	1
Malamute	1
Italian Mastiff	1
Boston Terrier	1
Pocket bulldog	1
Crossbreed*	1

## Discussion

The true burden of dog bites could be more enormous than data from hospital records. A community-based survey of 694 respondents in Cheshire, United Kingdom, revealed one in four people had experienced a dog bite, while only one-third of the victims sought medical care or intervention [14]. The incidence of dog bite-related hospital admissions has continued to rise in the last two decades. In England, the incidence rose from 6.34 to 14.99 admissions per 100,000 population from 1998 to 2018 [15]. This increase was driven by adults, which was also a similar phenomenon in Wales between 2014 and 2022 [16]. In Wales, children under age 14 comprised 20.8% of all dog bite and strike hospital admissions [16]. Ryan et al. reported a significant increase in the rate of emergency in-hospital admissions from 5.6 to 8.7 from 2012 to 2021 in Ireland, with highest rates among children under the age of 15 years [17]. Admissions in Scotland nearly tripled between 1997 and 2022 from 5.9 to 17.2 per 100,000 population [18].

Preschool children are predisposed to head and neck injuries from dog bites. [19]. They tend to crawl around dogs as they play. Seemingly benign child-dog interactions can result in head and face bite injuries [20]. These interactions are most often initiated by the child. Children under the age of two place their faces very close to moving objects, which may trigger a dog to react. The lack of danger awareness in very young children would increase the risk of dog attacks. Bite injuries to extremities were more likely seen in children aged over seven because of their increased height and limbs being in most proximity to the dog [19, 20]. In our study, three out of four children with bites on their lower limbs were over seven years old.

Most dogs involved in dog bite injuries are known to the victim. Up to 70% of dog bite cases involved family dogs, and incidents occurred mostly in home settings [21]. Zangari et al. reported 78% of the 127 cases were caused by family dogs [22] while Reisner et al. reported 72% of the 203 cases [20]. Our study reported 79.7% of the 172 cases, with 76.7% of the bites occurring in home settings. Older children were more likely to be bitten by unfamiliar dogs without interaction [20]. A population-based survey revealed 54.7% of people were bitten by unfamiliar dogs [14]. However, an increase in bites from familiar dogs was evident during the COVID-19 pandemic [6]. It is plausible that less severe dog bite injuries during the COVID-19 lockdown were managed at home due to anxiety around hospital attendance, thus underestimating the burden.

Dog bites can occur even in the presence of a responsible adult. Parents were mostly present (84%) when preschool children sustained dog bites [20]. This was like our study where 89% of bite injuries occurred in the presence of a responsible adult. Given that adult presence alone does not prevent dog bites, supervision requires more exploration and focus. Supervision of child-dog interaction is often deficient [23]. This was more evident during COVID-19 lockdowns probably due to parents struggling to meet conflicting demands.

Family dogs are mostly considered children’s companions and friends. Children love to pet, hug or stroke them. Dog body language can sometimes be misinterpreted. They find it challenging to recognize fearful dog behaviours [23]. Providing some guidance on child-dog interactions is a highly potentially protective factor against dog bites. Benign child-dog interactions such as kissing or hugging can provoke a dog bite [23]. In an observational study, parents were observed to be very attentive and stayed in proximity during interactions with unfamiliar dogs [24]. Such level of supervision with familiar dogs in daily lives is challenging to sustain. Children are left unattended around familiar dogs due to the inaccurate perception that children are not at risk. In the same vein, the lack of understanding that any dog can attack or bite humans under specific circumstances inhibits bite prevention [23]. There is unclear description of methods to improve adult supervision [25]. The National Institute of Healthcare Excellence (NICE) recommends the consideration of neglect when dog bite injury occurs in the context of inadequate supervision [25].

The evidence to support the correlation of specific dog breeds and bite injuries is limited and controversial [3]. Larger dogs may cause more severe injuries compared with small dogs because of relative differences in size. The XL bully breed was not responsible for the severe injuries in our cohort. Notably, small to medium-sized dog breeds inflicted some of the severe injuries in our cohort, highlighting that any dog is capable of causing life-changing injuries.

The psychological consequences on children and families have been understudied [26]. The impact is often poorly documented and hence underreported but can persist for years. Children with dog bite injuries may experience long-standing anxiety, phobia, nightmares, insomnia, social isolation or negative body image problems [27]. Ji et al. investigated 358 Chinese children with animal-induced injury and discovered important changes in their personalities, such as overcaution and cognitive constriction, which lasted for several years [28]. They reported psychological disturbances ranging from avoidance behaviour to formal diagnosis of post-traumatic stress disorder (PTSD). Nearly one-third of children developed phobia and avoidance of dogs while one-fifth experienced nightmares in a study by Boats et al. [26]. These injuries also have a significant impact on the victims' carers. Two-thirds of parents of children with dog bite injuries reported feelings of guilt and nearly half were apprehensive of their child’s safety [26].

There have been contentious debates regarding the effectiveness of breed-specific legislations in mitigating dog bite injuries. Much better animal welfare legislation is necessary to ensure better conditions for dogs so that they are less likely to bite people [29]. Most bites occur at home, so behaviour change is crucial. Adult supervision is vital for child injury prevention. Targeted education for dog owners regarding child-dog interactions is needed [30]. Modelling responsible behaviour around dogs by adults is pivotal in reducing the burden of dog bites. Raising awareness of appropriate behaviour around dogs, especially at homes, will reduce the risk of bite injuries. The effectiveness of integrating comprehensive programs into primary school curricula was demonstrated in a randomized controlled trial [31].

There are regional and socioeconomic patterns of dog bite injuries and hospital admissions with increased rates in most deprived areas [11, 18]. Therefore, targeting these areas with public health resources could maximize cost-effectiveness. Following these traumatic experiences, timely intervention to alleviate the psychological impact on children and families is essential for recovery.

Locally, we produced a user-friendly information sheet on safe child-dog interactions for pet lovers. The waiting area in the children's ED has a section where pictures of pets owned by ED staff are displayed. Copies of this information sheet were kept in this area for easy access to children and carers. A guide on appropriate antibiotic prescription was produced to reduce variation whilst supporting antimicrobial stewardship. To improve the quality of care for dog bite injuries, an electronic assessment template was launched in our ED with a clear emphasis on safeguarding, dog breed, incident logging with the police, and antibiotic prescription.

The study was limited by its retrospective nature, single-centre study, and some incomplete data. Selection bias was reduced by an independent review of compiled non-identifiable patient information prior to data collection. Electronic prescription went live after the study period, which contributed to incomplete data on pharmacological management. The study is strengthened by its originality of an under-researched subject in deprived areas in the UK. Although findings may not be generalizable, the study revealed data of public health importance, which strongly highlights the burden of dog bites in the paediatric population, and the significance of socioeconomic deprivation. Future research is necessary to understand the physical and psychological burden of paediatric dog bite injuries in the context of socioeconomic deprivation.

## Conclusions

Dog bites are preventable injuries of public health concern. Our study demonstrates the significant burden of these injuries. Younger children are much more likely to suffer severe injuries to the face and neck.

There are opportunities in the ED for parental education around adults' supervision. Importantly, our study highlights a probable disproportionate impact of dog bites on English regions with high indices of multiple deprivation. This buttresses the relevance of targeted interventions with community participation.
